# Glomerular filtration rate abnormalities in sickle cell disease

**DOI:** 10.3389/fmed.2022.1029224

**Published:** 2022-10-21

**Authors:** Nowah Afangbedji, Marina Jerebtsova

**Affiliations:** ^1^Department of Physiology and Biophysics, Howard University, Washington, DC, United States; ^2^Department of Microbiology, Howard University, Washington, DC, United States

**Keywords:** sickle cell disease, glomerular filtration, hyperfiltration, chronic kidney disease, sickle cell nephropathy, biomarkers

## Abstract

Sickle cell disease (SCD) is a group of inherited blood disorders affecting the β-globin gene, resulting in the polymerization of hemoglobin and subsequent sickling of the red blood cell. Renal disease, the most common complication in SCD, begins in childhood with glomerular hyperfiltration and then progresses into albuminuria, a fast decline of glomerular filtration, and renal failure in adults. This mini-review focuses on glomerular filtration abnormalities and the mechanisms of hyperfiltration, explores genetic modifiers and methods of estimating glomerular filtration rates, and examines novel biomarkers of glomerular filtration in SCD.

## Introduction

Sickle cell disease (SCD) is caused by a single nucleotide mutation in the β-globin gene resulting in the substitution of valine for glutamic acid leading to the polymerization of hemoglobin and sickling of red blood cells (RBCs) under low oxygen conditions. The pathophysiology of SCD is characterized by chronic hemolysis, vaso-occlusion, and organ damage. SCD may occur as homozygous hemoglobin S (HbSS), compound heterozygous inheritance of HbS with mutant hemoglobin C (HbSC), or mutations that result in decreased or absent β-globin (hemoglobin β+ and β0 thalassemia). HbSS and HbSβ0 genotypes are clinically similar disorders associated with severe anemia and disease complications, whereas HbSC and HbSβ+ thalassemia are relatively less severe. SCD is widespread in the malaria-endemic belt: sub-Saharan Africa; South America, the Caribbean, and Central America; Saudi Arabia; India; and the Mediterranean countries (1). It is also common among individuals whose ancestors came from these regions. Approximately 5% of the world’s population carries HbS mutation (2). The World Health Organization estimates that 300,000 children are born with SCD each year, 75% of whom are in sub-Saharan Africa, and this number could rise to 400,000 by 2050. In the USA, SCD affects approximately 100,000 people, primarily of African descent (3). Large-scale epidemiological studies demonstrated a strong geographical link between the highest HbS allele frequencies and high malaria endemicity on a global scale (1, 4–6).

Renal disease is one of the most common complications of SCD (2, 7). Approximately 30% of SCD patients develop chronic kidney disease (CKD), and 14–18% progress to end-stage kidney disease (8–10). Sickle cell nephropathy (SCN) is a progressive disease that begins in childhood as glomerular hyperfiltration leading to albuminuria, loss of renal function, and renal failure (11–15). Before the introduction of antibiotics prophylaxis in the 1970s, the largest mortality in SCD occurred during the first 5 years of life and was associated with bacterial infections (16). In adult patients, stroke and renal failure are common causes of death (16). Current improvements in patient care allow SCD patients to survive relatively longer, leading to an increased incidence of CKD and renal failure (17). Mortality due to renal failure in adult SCD patients is about 10.5% (10).

## Hyperfiltration in children and fast decline of glomerular filtration in adult sickle cell disease patients

Early studies demonstrated that glomerular changes in SCD patients are characterized by high renal blood flow and glomerular hyperfiltration and hypertrophy, starting in infancy and declining to a normal range during the first two decades of life (18–22). Glomerular hyperfiltration is defined as estimated glomerular filtration rate (eGFR) above 130 ml/min/1.73 m^2^ for women and 140 ml/min/1.73 m^2^ for men. There is no definition for hyperfiltration based on measured GFR (mGFR). The assessment of mGFR in 176 SCD infants demonstrated significantly higher values than in non-SCD infants (125.2 ± 34.4 in SCD vs. 91.5 ± 17.8 ml/min/1.73 m^2^ in controls). Schwartz formula for eGFR produced even higher values (184.4 ± 55.5 ml/min/1.73 m^2^) (22). Both measured and estimated GFR indicates increased prevalence (25–43%) of hyperfiltration in young SCD patients (12, 23–27), with the highest occurrence found in HbSS patients (28, 29).

In young SCD adult patients, hyperfiltration is common, particularly in early adulthood but declines into a normal range. The decline into a normal range is faster in men than women and represents a drop in renal function rather than an improvement. Hyperfiltration assessed by mGFR occurred in 51% of adult SCD patients, and the prevalence of hyperfiltration was higher in men (60%) than in women (42%) (28). We recently reported a high prevalence of hyperfiltration in a cohort of 51 adult SCD patients, with the highest occurrence in HbSS 67% and HbSC 50% patients (30), This result concords with a study of 193 SCD patients where the prevalence of hyperfiltration was 61% (13).

Glomerular enlargement and congestion, mainly in the juxtamedullary glomeruli, are more severe in children (31) than in adults, in whom glomerular scarring and fibrosis are developed (32). Hyperfiltration together with glomerular hypertrophy can induce glomerulosclerosis, leading to the reduction of GFR. GFR decline begins early and progresses more rapidly in individuals with SCD compared with the general population (33, 34).

A retrospective study over 4.01 years median follow-up in 331 patients demonstrated an annual eGFR decline of 2.05 ml/min/1.73 m^2^ for severe genotypes (HbSS and HbSβ0) and 1.16 ml/min/1.73 m^2^ for mild genotypes (HbSC and HbSβ+) (14). A higher rate of the annual eGFR decline (2.35 ml/min/1.73 m^2^) was observed by Xu et al. in 193 out of 288 SCD patients over the course of 5-years follow-up; a rate two-fold higher than in African American adults without SCD (13). The sex-associated renal decline suggests a higher and more common rate in men than women (13, 29). Even more, Asnani et al. found in their cohort an annual rate for mGFR decline in SCD patients to be even higher (3.2 ± 2.83 ml/min) (35).

The proportion of patients with fast eGFR decline was similar between patients with hyperfiltration and normal filtration, and eGFR values at baseline did not correlate with the rate of decline (13). Thus, the decline in eGFR is likely caused by intrinsic factors inducing the loss of renal function and does not reflect the difference in baseline glomerular filtration or improvement of hyperfiltration (13, 29). Because of hyperfiltration and higher GFR in young patients with HbSS and HbSβ0 genotypes, a longer time is needed to reach a GFR level of less than 60 ml/min/1.73 m^2^ that defines stage 3 CKD, and severe kidney injury may occur at higher GFR levels (14). Therefore, the rate of GFR decline may be a better predictor of progressive renal disease in SCD than the absolute value of eGFR.

## Mechanism of hyperfiltration in sickle cell disease

**FIGURE 1:**
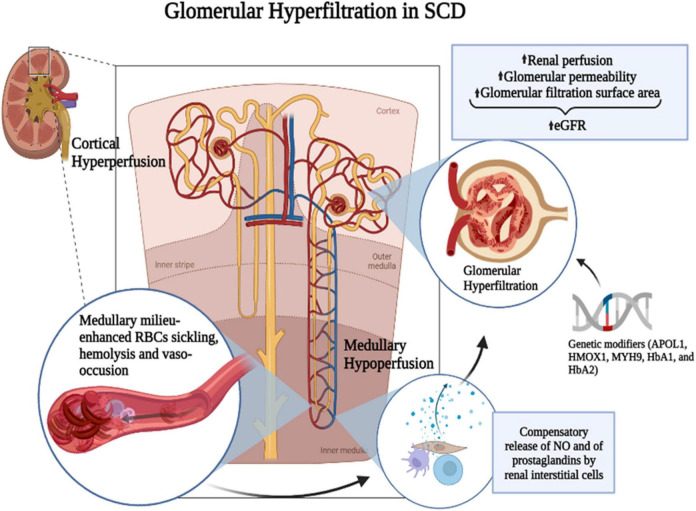
Mechanism of SCD-induced glomerular hyperfiltration. The hypoxic, acidic, and hyperosmolar environment of the renal medulla promotes the polymerization of HbS and the sickling of RBCs. Slow blood rheology in the vasa recta aggravates the vaso-occlusion and leads to ischemia that can advance to infarction. As a compensatory mechanism, medullary prostaglandins and nitric oxide (NO) are secreted leading to an increased renal cortical flow. It was suggested that prostaglandins are released by renal interstitial cells. Genetic modifiers such as APOL1, HMOX1, MYH9, HbA1, and HbA2 and increased renal perfusion coupled with increased glomerular permeability and glomerular filtration surface area may enhance glomerular hyperfiltration in SCD. Figure created with BioRender.com.

Hyperfiltration and renal injury in SCD result from a cascade of events starting with chronic RBC sickling and hemolysis, leading to increased blood viscosity, microvascular obstruction, anemia, oxidative stress, and inflammation (36). The most common histopathological change in the SCD kidney is the dilation of glomerular and interstitial capillaries filled with sickle RBCs (37). The mechanism leading to hyperfiltration is associated with enhanced renal blood flow (20, 25, 38, 39), reflecting an increased cardiac output due to anemia and indicated by the positive correlation of hyperfiltration with low hemoglobin levels (40, 41). SCD is characterized by a paradoxical coexistence of hypoperfusion in the microvasculature and a hyperperfusion observed systemically. The kidney exemplifies this paradox with enhanced perfusion in the whole kidney and the glomeruli while the vasa recta remain hypoperfused. The hypoxic, acidic, and hyperosmolar environment of the renal medulla promotes the sickling of RBCs and slow blood rheology. In the vasa recta, slow blood rheology aggravates vaso-occlusion and leads to ischemia that can advance to infarction. As a compensatory mechanism, medullary prostaglandins and nitric oxide are secreted, leading to an increased renal cortical flow (42–44) (Figure 1). A screening of 14 HBSS patients using inulin, paraaminohippuric acid, and dextran clearances demonstrated increased renal perfusion and a 65% increase in ultrafiltration coefficient (Kf) in SCD patients compared to non-SCD controls (25). An elevated Kf suggests an increased glomerular filtration area and supports glomerular enlargement in SCD. Administration of prostaglandins synthesis inhibitor indomethacin in SCD patients significantly reduced GFR (45). Indomethacin inhibits cyclo-oxygenase (COX), which is required for the synthesis of prostaglandins. However, non-steroidal anti-inflammatory drugs (NSAIDs) are associated with renal, gastrointestinal, and cardiovascular toxicity (46). In the general population, NSAIDs were associated with CKD and a rapid decline in GFR (47–49). In pediatric SCD cohorts, NSAIDs increased the risk of micro-albuminuria and, in one reported case, irreversible renal failure (50, 51). Thus, the use of prostaglandins inhibitors to reduce GFR is more detrimental than beneficial for SCD patients.

Additionally, intravascular hemolysis reduces NO bioavailability through binding to plasma-free hemoglobin and activation of arginase. Several studies demonstrated that markers of chronic hemolysis, such as low total hemoglobin and fetal hemoglobin levels, correlate with hyperfiltration (28, 52, 53). On the contrary, in a transgenic SCD mouse model, nitric oxide synthase 2 (iNOS) induction was shown in the glomeruli and distal nephron. Elevation of iNOS may reflect a feedback mechanism activated by low NO, but it increases NO synthesis, leading to vasodilation that contributes to hyperfiltration (54).

Sickle cell disease increases inflammation and oxidative stress, which induce endothelial injury and glomerulopathy. We and others demonstrated that in the mouse model, SCD nephropathy is associated with glomerular endothelial inflammation induced by activation of the endothelin and RON kinase receptors (37, 55). Treatment with endothelin receptor antagonist or RON kinase inhibitor significantly ameliorated glomerular endothelial injury and hypertrophy. Additionally, statin (atorvastatin) treatment improved urine concentrating ability and glomerular filtration rate, decreased endothelial activation markers, and ameliorated oxidation stress (56). In a pilot study involving 13 SCD patients, treatment with atorvastatin resulted in a small increase in eGFR (57). The protective effects of statins may be due to their anti-inflammatory and antioxidant function.

There is also some evidence that glomerular permeability is increased in SCD, caused by a change in the glomerular porosity: changes in the number of glomerular membrane pores, the size, and the selectivity of the pores. Such findings may suggest a unique, additional, non-hemodynamic-related cause of hyperfiltration in SCD (39).

High plasma glucose and blood pressure (BP) levels are associated with hyperfiltration in diabetic and obese patients (58, 59). In contrast, SCD patients have normal glucose regulation and low BP (25, 26, 38). The incidence of hypertension in patients with HbSS is significantly lower than in non-SCD African Americans in the USA (2–6% in SCD vs. 28% in non-SCD). The slightly increase in BP values considered normal in non-SCD individuals may be a risk factor for cardiovascular complications in SCD patients (60). In the general population, obesity is a risk factor for the progression of renal disease. However, in SCD patients, a higher body mass index is associated with decreased odds of rapid eGFR decline (21, 61).

## Genetic modifiers of renal disease in sickle cell disease

Plasma level of fetal hemoglobin (HbF) is a best-characterized modifier of SCD that negatively correlates with the severity of complications. Low HbF levels are associated with more hemolysis (62). Single nucleotide polymorphisms in the promoters of gene encoding HbF, BCL11A, and HMIP-2 are strongly associated with increased HbF levels (63, 64). Hydroxyurea treatment increases HbF levels (65, 66) and is recommended for almost all patients with HbSS and HbSβ0 disease starting in the first year of life. However, high levels of endogenous HbF or hydroxyurea treatment do not significantly improve GFR or reduce hyperfiltration (67). Multiple genetic modifiers, including *APOL1, HMOX1, MYH9, HbA1*, and *HbA2* variants, are implicated in the development and progression of SCN (33, 68–70). The results of these studies are not consistent. Interestingly, data analysis of 326 SCA patients from the Democratic Republic of Congo indicated that *APOL1* high-risk genotypes (G1/G1, G2/G2, and G1/G2) were significantly associated with hyperfiltration, but HMOX1 GT-dinucleotide long repeats were associated with lower eGFR (71). In contrast, a cohort of 521 African American SCD patients only demonstrated a weak correlation between MYH9 and APOL1 genotype and eGFR (72).

## Estimated glomerular filtration rate calculation methods

Currently, no consensus exists regarding the optimal eGFR equation for SCD patients (73, 74). The evaluation of eGFR with formulas based on the serum creatinine and/or cystatin C concentration is unreliable for SCD patients because of the increased glomerular filtration, lower muscle mass, and increased tubular secretion of low molecular mass organic molecules (25, 36, 75, 76). Plasma creatinine is affected by physiological and analytical factors, and the extreme deviation of eGFR from mGFR is frequent. Non-SCD African Americans also have higher serum creatinine levels and urinary creatinine excretion than the general population (74).

In children with SCD, Schwartz equation based on height and serum creatinine is commonly used for eGFR calculation. A study in 176 HbSS infants found no correlation between mGFR and eGFR estimated by Swartz equation (22). Additionally, Schwartz estimation is highly dependent on the accuracy of the creatinine detection (77). A study in 79 SCD children demonstrated that eGFR calculation based on creatinine detected by mass-spectrometry is better correlated with mGFR (77).

Several equations for eGFR have been used for adult SCD patients, including Cockcroft–Gault (CG), the Modification of Diet in Renal Disease (MDRD), and Chronic Kidney Disease Epidemiology-Collaboration (CKD-EPI) equations with and without adjustment for race (73, 78–80). A study in 59 adult SCD patients demonstrated that CG and MDRD formulas overestimated creatinine clearance compared to mGFR (78). Similar results were obtained in 48 adult HbSS patients, where 66% of the cohort had hyperfiltration assessed by mGFR compared to 72% using MDRD formula (28, 79). The serum creatinine-based CKD-EPI equation performed relatively well but produced a systematic bias of about 45 ml/min/1.73 m^2^ (79). The use of race for eGFR calculation produces an additional bias because SCD predominantly affects Africans and African Americans and, equations with adjustment for the black race overestimate eGFR (73, 79). New eGFR equations based on creatinine and cystatin C without race have recently been developed (80). The new equations slightly underestimate eGFR in African Americans and overestimate it in the general population. The new creatinine-cystatin C equations without race were more accurate than new creatinine equations, with smaller differences between race groups (21, 80). Also, because the tubular secretion is the predominant mode of creatinine excretion when the GFR is less than 40 ml/min/1.73 m^2^, serum creatinine levels will produce false values in assessing GFR in older SCD patients with reduced metabolism and tubular dysfunction (36, 76, 81, 82). Thus, the absolute values of eGFR have limited efficacy for routine evaluation of GFR in SCD children and adult patients.

## Markers of renal function in sickle cell disease

Glomerular hyperfiltration in SCD plays an essential role in the progression of renal disease; however, to date, no reliable biomarkers of hyperfiltration exist (78). Common clinical markers of renal function, such as serum creatinine and cystatin C are unreliable in estimating GFR in SCD. Reduced production and increased clearance of plasma creatinine may lead to falsely normal plasma creatinine levels and creatine clearance, and delay the detection of kidney disease. Direct measurement of GFR with the injection of substances that do not undergo metabolism, tubular secretion, and absorption, such as inulin, iohexol, iothalamate, and 51Cr-EDTA is recommended; however, the method is difficult and rarely used in the clinical setting. Recently, several potential biomarkers of glomerular filtration were assessed.

Chronic sickling and hemolysis of RBCs induce multiple mechanisms that cause kidney injury. Thus, hemolysis markers such as hemoglobin (Hb), bilirubin, lactate dehydrogenase (LDH) and reticulocyte, and RBCs Hb levels were explored. Studies provided conflicting evidence of the correlation between GFR and hemolysis: Some studies did not find a significant relationship (62, 66, 83), but others showed a strong correlation (13, 28, 83). In a large patient cohort study of 356 patients from the University of Illinois Chicago and 439 patients from the multi-center cohort, Saraf et al. demonstrated an association of hemoglobinuria with progressively lower eGFR values (83). Using a multiple logistic regression analysis in 280 adult SCA patients, Haymann et al. found that hyperfiltration is independently associated with lower HbF and total Hb levels (28). In contrast, in the Kuwaiti SCD patients, Marouf et al. did not find a correlation between eGFR and HbF concentration (78). Higher platelet and reticulocyte counts, higher systolic BP, lower Hb level, and body mass index were associated with a rapid decline of GFR in the study of 288 adult SCD patients (13). And, though BP levels in SCD are lower than in a general population of African Americans, GFR does not correlate with BP (84).

Metabolomics analysis identified two metabolites (asymmetric dimethylarginine – ADMA and quinolinic acid) associated with a rapid decline of GFR (13). ADMA is the endogenous nitric oxide (NO) synthase inhibitor implicated in the pathogenesis of endothelial dysfunction and CKD progression (85). Quinolinic acid was previously associated with incident CKD in the general population (86).

In our study, mass-spectrometry analysis of urine samples collected from HbSS patients identified orosomucoid (ORM) (87) and ceruloplasmin (CP) (88) which positively correlated with hemoglobinuria, and kringle domain-containing protein HGFL, which positively correlated with GFR (89). ORM is a major acute-phase protein and increased ORM expression and serum concentration is associated with tissue injury, inflammation, infection, cancer, and diabetic and systemic lupus erythematosus-associated renal disease (90, 91). CP is a ferroxidase that plays a central role in iron homeostasis, which is highly affected by SCD. Urinary CP was proposed as a biomarker for early diagnosis of membranous nephropathy, focal segmental glomerulosclerosis, lupus nephritis, and IgA nephropathy (92, 93). Therefore, hemoglobinuria, CP, and ORM reflect increased inflammation and iron metabolism in SCD patients and positively correlate with stages of CKD. Plasma and urine ORM inversely correlated with eGFR in HbSS and HbSC patients (30). Despite considerable efforts in searching for SCN-associated biomarkers, there remains a paucity of reliable biomarkers of GFR in SCD.

## Conclusion

In summary, SCN is a common cause of morbidity and mortality in SCD patients (36, 94, 95). Despite severe complications of SCD, it remains the most common inherited hemoglobinopathy due to positive environmental selection. The mechanism of renal disease in SCD is unique (39). The higher GFR in patients with albuminuria is consistent with the hypothesis that high glomerular flows cause renal damage (38, 73). But not all hyperfiltrating SCD patients develop albuminuria and renal disease, suggesting that other mechanisms and genetic risk factors are involved in the disease development and progression. More studies are needed to identify novel mechanisms and consistent genetic factors associated with SCN. Common clinical markers of renal function, such as serum creatinine and cystatin C, are unreliable because of increased GFR, low muscular mass, and increased tubular secretion in SCD patients (28, 75). Although an accurate measure of GFR is not necessary for most clinical purposes, the consequences of eGFR overestimation are extremely important in rapid progressors who may benefit from early treatment. Thus, monitoring of eGFR during routine clinical visits may reveal a fast decline of eGFR better than other markers predicting the development of renal disease in SCD patients (96).

## Author contributions

NA and MJ wrote the manuscript. Both authors contributed to the article and approved the submitted version.
